# Types of Physical Activity in Nonalcoholic Fatty Liver Disease and All-Cause and Cardiovascular Mortality

**DOI:** 10.3390/jcm12051923

**Published:** 2023-02-28

**Authors:** Donghee Kim, Karn Wijarnpreecha, Brittany B. Dennis, George Cholankeril, Aijaz Ahmed

**Affiliations:** 1 Division of Gastroenterology and Hepatology, Stanford University School of Medicine, Stanford, CA 94305, USA; 2 Division of Gastroenterology and Hepatology, Department of Medicine, University of Arizona College of Medicine, Phoenix, AZ 85004, USA; 3 Division of Gastroenterology and Hepatology, Department of Internal Medicine, Banner University Medical Center, Phoenix, AZ 85006, USA; 4 British Columbia Centre on Substance Use, University of British Columbia, Vancouver, BC V6Z 2A9, Canada; 5 Liver Center, Division of Abdominal Transplantation, Michael E. DeBakey Department of General Surgery, Baylor College of Medicine, Houston, TX 77030, USA; 6 Section of Gastroenterology and Hepatology, Department of Medicine, Baylor College of Medicine, Houston, TX 77030, USA

**Keywords:** exercise, hepatic steatosis, lifestyle modification, NHANES, death

## Abstract

The impact of different types of physical activity (PA) on mortality in the context of nonalcoholic fatty liver disease (NAFLD) is not clearly defined and was investigated. This prospective study was performed using the 2007–2014 US National Health and Nutrition Examination Survey with mortality follow-up through 2019. Over a median follow-up of 8.6 years, leisure-time and transportation-related PA that fulfilled the criteria outlined in the PA guidelines (≥150 min/week) in NAFLD were associated with a risk reduction in all-cause mortality (hazard ratio [HR]: 0.76, 95% confidence interval [CI]: 0.59–0.98 for leisure-time PA; HR: 0.62, 95% CI: 0.45–0.86 for transportation-related PA). Leisure-time and transportation-related PA in NAFLD were inversely associated with all-cause mortality in a dose-dependent manner (*p* for trends <0.01). Furthermore, the risk for cardiovascular mortality was lower in those meeting the PA guidelines for leisure-time PA (HR: 0.63, 95% CI: 0.44–0.91) and transportation-related PA (HR: 0.38, 95% CI: 0.23–0.65). Increasing sedentary behavior was linked to an increased risk of all-cause and cardiovascular mortality (*p* for trend <0.01). Meeting PA guidelines (≥150 min/week) for leisure-time and transportation-related PA has beneficial health effects on all-cause and cardiovascular mortality among individuals with NAFLD. Sedentary behavior in NAFLD showed harmful effects on all-cause and cardiovascular mortality.

## 1. Introduction

Nonalcoholic fatty liver disease (NAFLD), the world’s most prevalent chronic liver disease, affects a third of the United States (US) population [1]. NAFLD consists of a clinicopathologic spectrum ranging from nonalcoholic fatty liver and nonalcoholic steatohepatitis to end-stage liver disease, including cirrhosis and hepatocellular carcinoma [2,3]. On top of its enormous existing disease burden, NAFLD is projected to demonstrate an exponential growth in its prevalence in the next decade. In addition, the risk of progression to end-stage liver disease, combined with an increased risk of co-morbid cardiovascular disease [4] and an unapproved pharmacological treatment, makes clinical management extremely difficult. Individuals with NAFLD are treated with lifestyle modifications, which are challenging to implement. NAFLD is closely associated with individual components of the metabolic syndrome, including diabetes, dyslipidemia, and abdominal obesity, which also benefit from physical activity (PA) [5]. PA is a pivotal determinant of lifestyle modifications and is commonly recommended for individuals with NAFLD, usually alongside dietary control and weight loss. Several studies have demonstrated an inverse association between PA and NAFLD [6,7,8]. However, most studies have focused on leisure-time PA. Additionally, guidelines for the management of NAFLD mainly focused on weight loss and diet, while the types, intensity, and amount of PA needed for optimal therapeutic effects in NAFLD were unclear [2,3]. Sedentary behavior was also associated with diabetes and cardiovascular disease, which were closely associated with NAFLD [9]. Therefore, education on reversing sedentary behavior PA among individuals with NAFLD may provide an additional therapeutic option. Although there is robust observational evidence for the beneficial effects of PA on the risk of NAFLD [6,8,10,11], the impact of PA on mortality in individuals with NAFLD is not well determined. A recent study using accelerometer-assessed PA reported that increasing PA is associated with lower all-cause and cardiovascular mortality in individuals with NAFLD [12]. Although a recent study showed that leisure-time PA and transportation-related PA have a significant dose-dependent protective effect on NAFLD [10], there is little evidence determining whether the types and amounts of PA beneficially affect all-cause mortality and cardiovascular mortality among individuals with NAFLD. The 2018 Physical Activity Guidelines for Americans recently outlined the amounts and types of PA that provide significant health benefits [13]. For substantial health benefits, these guidelines recommend adults be physically active for at least 150 to 300 min per week of moderate intensity [13]. This recommendation emphasizes that reversing sedentary behavior will benefit nearly everyone [13]. Therefore, we studied whether the types of PA meeting guidelines and the amount of PA among individuals with NAFLD are associated with all-cause and cardiovascular mortality in a nationally representative sample of the United States.

## 2. Materials and Methods

### 2.1. Subjects and Study Design

We performed analyses of the recent four 2-year waves of the National Health and Nutrition Examination Survey (NHANES) 2007–2014, with follow-up for at least 5 years through 31 December 2019, to estimate mortality in the study cohort. A stratified, multi-staged, and clustered probability sampling design was employed to provide a nationally representative sample of non-institutionalized civilians in the US.

A total of 22,673 adults (≥20 years of age) were examined for laboratory tests at a mobile examination center. We excluded 3173 individuals that had hepatitis B virus infection (determined by the presence of the hepatitis B surface antigen), hepatitis C virus infection (determined by the presence of hepatitis C antibody), significant alcohol consumption (>30 g/day in men and >20 g/day in women), were pregnant, those with incomplete or missing data on PA questionnaire and/or serum aminotransferase, body mass index (BMI), and mortality status, and those that had been exposed to a medication with a known association to fatty infiltration of the liver (i.e., amiodarone, corticosteroid, methotrexate, tamoxifen, and valproate) for more than 6 months. The first study sample consisted of 10,853 individuals with NAFLD, as defined by the Hepatic Steatosis Index (HSI) [14]. The second study sample included 3263 individuals with NAFLD as defined by the US Fatty Liver Index (USFLI) [15], and was created from the individuals that underwent laboratory tests after a fast of at least 8 hours from the first study sample.

The National Center for Health Statistics’ Research Ethics Review Board has reviewed and approved this original NHANES, and all individuals signed a full informed consent. Since de-identified data was used in the study, this analysis was exempted by the Institutional Review Board.

### 2.2. Clinical and Laboratory Evaluations

Methods used for clinical and laboratory evaluations have been described in detail elsewhere [10]. Briefly, the NHANES 2007–2014 consisted of sociodemographic information, anthropometric measures, comprehensive questionnaires, and laboratory tests. We categorized race/ethnicity as non-Hispanic white, non-Hispanic black, Hispanic (Mexican-American or Other Hispanic), or others. Marital status was dichotomized as being married or cohabitating with a partner versus others. Educational status was defined as lack of high school graduation versus high school graduation. The individuals’ family income-to-poverty ratio classified the economic situation as either ≤0.99 or ≥1.00 (at or above poverty). We defined hypertension as having a systolic blood pressure of ≥140 mmHg or a diastolic blood pressure of ≥90 mmHg and/or being on anti-hypertensive medication. We defined diabetes mellitus as fasting plasma glucose levels of ≥126 mg/dL and/or the use of a hypoglycemic agent or insulin.

### 2.3. Physical Activity and Sedentary Behavior Questionnaire

Methods used for PA and sedentary behavior have been described in detail elsewhere [10]. In brief, all participants answered a questionnaire modeled after the Global Physical Activity Questionnaire. The types of PA assessed were leisure-time, occupation, and transportation-related PA. Each type of PA included questions detailing intensity (vigorous vs. moderate), frequency (per week), and duration (minutes) in a typical week. Individuals provided details and a breakdown of vigorous versus moderate-intensity PA during occupation and leisure time. As previously validated, minutes of vigorous PA were doubled and added to moderate PA minutes for occupation-related and leisure-time PA [16]. The amount of total PA was estimated by summing up leisure-time PA, occupation-related PA, and transportation-related PA. According to the 2018 Physical Activity Guidelines for Americans (adults engage in ≥150 min/week of moderate-intensity PA, 75 min/week of vigorous-intensity PA, or an equivalent combination) [13]. We categorized PA as physically inactive (“not meeting PA guidelines”) in those who did not meet the criteria for the 2018 Physical Activity Guidelines for Americans and physically active (“meeting PA guidelines”) in those who met the 2018 Physical Activity Guidelines for Americans. We investigated sedentary behavior as total sitting time, which was recorded in hours per day in a typical week.

### 2.4. Definition of NAFLD

To define NAFLD, we used two previously well-validated non-invasive panels for fatty liver [14,15,17,18,19]. In the absence of other causes of chronic liver disease, significant alcohol consumption, and use of steatogenic medication, NAFLD was defined using the HSI and the USFLI. We calculated HSI by using the following equation: HSI = 8 × (alanine aminotransferase/aspartate aminotransferase ratio) + BMI (+2, if diabetes; +2, if female) [14]. We used the published cut-off of 36 to define the presence of NAFLD [14]. As the USFLI equation requires fasting glucose and insulin, analyses using the USFLI included a subgroup of individuals examined after a minimum fast of 8 h. We calculated the USFLI using the following equation: USFLI = (e^−0.8073 × non-Hispanic black + 0.3458 × Mexican American + 0.0093 × age + 0.6151 × loge (gamma-glutamyl transferase) + 0.0249 × waist circumference + 1.1792 × loge (insulin) + 0.8242 × loge (glucose) − 14.7812^)/(1+ e^−0.8073 × non-Hispanic black + 0.3458 × Mexican American + 0.0093 × age + 0.6151 × loge (gamma-glutamyl transferase) + 0.0249 × waist circumference + 1.1792 × loge (insulin) + 0.8242 × loge (glucose) − 14.7812^) × 100 [15]. Advanced fibrosis was defined as having at least one of the high probabilities for advanced fibrosis calculated using three non-invasive fibrosis panels (the NAFLD fibrosis score, the fibrosis-4 score, and the aspartate aminotransferase-to-platelet ratio index) [17].

### 2.5. Mortality

All individuals over 20 years in the NHANES 2007–2014 had passive mortality follow-up through 31 December 2019 [20]. For decedents, an enhanced linkage algorithm was designed to assess mortality status by date of death and cause of death based on the National Death Index. The leading cause of death was coded as the Underlying Cause of Death 113 (UCOD_113) code. All-cause mortality and the following two cause-specific mortalities were defined as cardiovascular disease (UCOD_113: 55–68, 70) and cancer (UCOD_113: 19–43).

### 2.6. Statistical Analysis

Due to the complex survey design employed by the NHANES, we used appropriate sample weights, stratification, and clustering to obtain representative US population-level data. The weighted frequencies (95% confidence intervals [CI]) and the weighted means ± standard errors were presented appropriately. The chi-square test for categorical variables or linear regression for continuous variables was used. We used the weighted Cox proportional hazards regression analysis for survival, including all-cause and cause-specific mortality. Multivariable weighted Cox proportional models were performed to assess the independent association of types of PA and sedentary behavior with all-cause and cause-specific mortalities after considering other potential demographic and clinical confounders. We performed all analyses using STATA 17.0 (Stata Corp., College Station, TX, USA) using Taylor series linearization.

## 3. Results

We performed analyses using 10,853 individuals with NAFLD (mean age, 47.9 years; 48.3% males). As shown in Table 1, there were noticeable differences in the clinical characteristics of individuals with NAFLD based on their PA status. When compared to physically inactive individuals with NAFLD, physically active individuals with NAFLD were more likely to be younger, educated, above the poverty level, men, and less likely to have diabetes and/or hypertension. Physically active individuals with NAFLD also had a lower BMI and waist circumference, fasting glucose levels, hemoglobin A1c, high-density lipoprotein-cholesterol, and higher aminotransferase levels than physically inactive individuals with NAFLD.

During a median follow-up period of 8.6 years (interquartile range: 6.5–10.8 years), 1111 deaths (369 from cardiovascular disease and 272 from cancer) were reported among individuals with NAFLD. Results of Cox-regression analyses are summarized in Table 2. Total PA that met the criteria outlined by PA guidelines (≥150 min/week) was independently associated with a 26% lower risk of all-cause mortality (hazard ratio [HR]: 0.74, 95% CI: 0.60–0.90). When we sub-analyzed each type of PA, leisure-time PA fulfilling the PA guidelines (≥150 min/week) was associated with a reduction in the risk of all-cause mortality in the age- and sex-adjusted model (HR: 0.66, 95% CI: 0.52–0.84). In the multivariable model adjusted for known demographic variables and traditional risk factors, leisure-time PA meeting the criteria of PA guidelines demonstrated a 24% lower hazard for all-cause mortality. Transportation-related PA that met PA guidelines (≥150 min/week) was associated with a lower risk for all-cause mortality in the age- and sex-adjusted model (HR: 0.62, 95% CI: 0.46–0.85) and in the multivariable model (HR: 0.62, 95% CI: 0.45–0.86). The addition of waist circumference to the model did not change the HRs significantly for leisure-time and transportation-related PA. Occupation-related PA showed no association with all-cause mortality in age- and sex-adjusted models or in multivariable models.

To assess whether there is a dose-response relationship between different types of PA and all-cause mortality and to further assess the impact of various levels of PA above and below the recommended PA guidelines, we categorized PA into four levels of intensity based on the duration: 0, <150, 150–299, ≥300 min/week (Table 3). Similar beneficial effects on survival were also observed across different total PA categories. When adjusted for multiple confounders, individuals performing <150 min/week of leisure-time PA had a 29% lower risk of all-cause mortality (HR: 0.71, 95% CI: 0.55–0.90) compared to physically inactive individuals. Those who reported 1–2 times (150–299 min/week) and over 2 times (≥300 min/week) the recommended level of leisure-time PA had a 32% (HR: 0.68, 95% CI: 0.48–0.97) and a 26% (HR: 0.74, 95% CI: 0.56–0.98) lower risk of all-cause mortality, respectively. Transportation-related PA was also inversely associated with all-cause mortality in dose-dependent manners (*p* for trend = 0.002) in the multivariable model. When we adjusted for waist circumference, the inverse dose-response association between total, leisure-time, or transportation-related PA and all-cause mortality was attenuated but maintained (*p* for trend < 0.01). However, there was no significant dose-response relationship between occupation-related PA and all-cause mortality except for individuals performing 1–300 min/week. When we performed sensitivity analyses using the USFLI (Supplementary Tables S1 and S2), adjusting for age and sex, and in multivariable models, we observed a similar and significant association of total and leisure-time PA with all-cause mortality. We performed sensitivity analyses to determine the impact of diabetes-related NAFLD or obesity-related NAFLD on all-cause mortality compared to their counterparts, respectively (Supplementary Table S3). We found that NAFLD with diabetes- or obesity-related NAFLD was associated with an increased risk for all-cause mortality. Similar and significant associations were shown between leisure-time or transportation-related PA and all-cause mortality. After adjusting for advanced fibrosis (Supplementary Table S4), sensitivity analysis revealed comparable significant associations between leisure-time or transportation-related PA and all-cause mortality. We found advanced fibrosis to be an independent risk factor for all-cause mortality. When the analysis was stratified by advanced fibrosis in NAFLD (Supplementary Table S5), results were largely identical except for the significant association between occupation-related PA and all-cause mortality.

When the analysis was restricted to cardiovascular mortality (Table 4 and Table 5), there was an association with a lower risk for cardiovascular mortality in those meeting PA guidelines for total PA (HR: 0.65, 95% CI: 0.49–0.87), leisure-time PA (HR: 0.63, 95% CI: 0.44–0.91), and transportation-related PA (HR: 0.38, 95% CI: 0.23–0.65), and this association remained significant after adjusting for waist circumference. As shown in Table 5, multivariable analyses showed a dose-dependent relationship between the degree of leisure-time or transportation-related PA and cardiovascular mortality (*p* for trend = 0.001). In particular, individuals with over two times (≥300 min/week) the recommended level of activity per PA guidelines for leisure-time PA or transportation-related PA demonstrated a 66% lower risk of cardiovascular mortality (HR: 0.34, 95% CI: 0.21–0.55) and 72% (HR: 0.28, 95% CI: 0.14–0.55). Our results were largely identical when we performed sensitivity analyses using USFLI (Supplementary Tables S6 and S7) in age- and sex-adjusted and multivariable models. When we performed the same analyses among individuals without NAFLD (Supplementary Tables S8–S10), the overall results remained similar: the protective effect of leisure-time PA on all-cause and cardiovascular mortality was slightly higher than estimates in those with NAFLD, but the protective effect of transportation-related PA on all-cause and cardiovascular mortality was statistically insignificant. Instead, increasing occupation-related PA significantly influenced all-cause mortality (HR 0.79, 95% CI 0.66–0.95) among individuals without NAFLD. In terms of cancer-related mortality, there was no association between various types of PA in NAFLD and cancer-related mortality in age- and sex-adjusted and multivariable models (Supplementary Table S11). Notably, we found that total PA meeting the criteria of PA guidelines demonstrated a 46% lower hazard for cancer-related mortality among individuals without NAFLD (HR: 0.54, 95% CI: 0.38–0.78, Supplementary Table S12).

Increasing sedentary behavior was dose-dependently associated with increased risk for all-cause mortality and cardiovascular mortality in the age- and sex-adjusted and multivariable model (*p* for trend < 0.001, Table 6). When we considered total PA and sitting time simultaneously, the dose-response association of sitting time with all-cause and cardiovascular mortality was slightly attenuated but maintained (*p* for trend < 0.01). Over 8 h of sitting time, irrespective of different total PA categories, had a 27% (HR: 1.27, 95% CI: 1.06–1.53) and 62% (HR: 1.62, 95% CI: 1.10–2.39) higher risk of all-cause and cardiovascular mortality, respectively. When the analysis was repeated using the USFLI formula to define NAFLD, the results were largely identical but changed insignificantly with wide CIs (Supplementary Table S13). Similarly, increasing sedentary behavior was associated with increased risk for all-cause mortality and cardiovascular mortality among individuals without NAFLD (Supplementary Table S14).

## 4. Discussion

While strong evidence supports the beneficial effects of specific types of PA on NAFLD [10,11], the longitudinal association of different types of PA with all-cause and cardiovascular mortality has not been sufficiently investigated among individuals with NAFLD. In this prospective population-based study, we noted that fulfilling PA guidelines for leisure-time and transportation-related PA in individuals with NAFLD was associated with lower all-cause and cardiovascular mortality. Leisure-time and transportation-related PA had a significant dose-dependent protective effect on all-cause and cardiovascular mortality, independent of known coexisting risk factors. In addition, we noted a harmful effect of sedentary behavior on survival independent of total PA among individuals with NAFLD.

There is a scarcity of literature on the association between different types of PA and mortality in individuals with NAFLD. Previous studies on the effects of PA on NAFLD have investigated the severity or development of NAFLD rather than all-cause and cause-specific mortality [6,7,21]. A Norwegian study showed the survival benefit of an estimated high cardiorespiratory fitness by a prediction model in NAFLD [22]. A recent study determined that increasing total cumulative PA in NAFLD benefits all-cause mortality, which may be partly derived from a reduction in cardiovascular mortality [12]. Consistent with these studies, our study found that increasing total PA among individuals with NAFLD was associated with a lower risk for all-cause and cardiovascular mortality in a nationally representative sample. In comparison to other above-mentioned studies, our study has the advantage of demonstrating an association of specific types of PA and meeting the criteria for PA guidelines with all-cause and cardiovascular mortality. We observed significant benefits for leisure-time and transportation-related PA for recommended PA on all-cause mortality and an additional benefit of more than twofold for the minimum recommended PA on cardiovascular mortality. The likelihood of meeting PA guidelines was lower in individuals with NAFLD than in those without NAFLD [10]. In the absence of an approved pharmacologic treatment for NAFLD, our study found that meeting PA guidelines (>150 min/week) for leisure time and transportation-related PA improved survival in individuals with NAFLD. While we observed that advanced fibrosis and NAFLD co-existing with diabetes or obesity were independent risk factors for all-cause mortality, the independent association of leisure-time and transportation-related PA with all-cause mortality was also noted. Therefore, we suggest that specific types of PA for >150 min/week be integrated as lifestyle modifications into the clinical management plan of patients with NAFLD to provide a survival benefit.

This study did not show the beneficial effect of any type of PA on cancer-related mortality among individuals with NAFLD, although there was a beneficial effect of total PA on cancer-related mortality among individuals without NAFLD, consistent with a previous study [12]. The putative mechanistic pathways may include improvements in insulin sensitivity, reduction of visceral fat, lowering the level of carcinogenic adipocytokines, etc. [23]. This discrepancy may be explained by the small sample size due to the relatively short follow-up period. In addition, it has been observed that the association of PA with cancer-related mortality differs depending on the type of cancer [24]. While significant inverse associations were observed for colorectal and breast cancer-related mortality, which may be more prevalent among individuals without NAFLD [24], a few studies have reported an inverse association for liver cancer-related mortality, which was more prevalent among individuals with NAFLD [25]. Moreover, it may be more challenging to achieve decreased visceral fat, improved insulin sensitivity, and decreased adipokines among individuals with NAFLD than those without NAFLD [12]. Future studies are needed to determine these observations.

A previous study showed a significant decline in the frequency of meeting the criteria for transportation-related PA during 2007–2016 for individuals with NAFLD compared to those without NAFLD in the United States [10]. In this study, transportation-related PA significantly impacted all-cause and cardiovascular mortality only among individuals with NAFLD. Therefore, clinicians may recommend individuals with NAFLD to engage in transportation-related PA, beyond leisure-time PA, to improve health outcomes.

The 2018 Physical Activity Guidelines for Americans recently addressed sedentary behavior and its harmful effects on health [13]. Given its high prevalence among individuals with NAFLD, sedentary behavior can be considered an important therapeutic target in treating individuals with NAFLD. Our study is the first to report that increasing sedentary behavior among individuals with NAFLD was dose-dependently associated with increased risk for all-cause mortality and cardiovascular mortality. In the two previous studies assessing the association between sedentary behavior and mortality in the setting of NAFLD [12,22], sedentary behavior was not associated with an increased risk of all-cause and cardiovascular mortality compared with those with non-sedentary behavior. The current 2018 Physical Activity Guidelines for Americans do not specify a time threshold for sedentary behavior [13]. Our study has clearly demonstrated that over 8 h of sitting time has a higher risk of all-cause and cardiovascular mortality, irrespective of total PA categories. Therefore, clinicians may need to educate physically inactive individuals with NAFLD about the health benefits of avoiding sedentary behavior to improve survival.

The strengths of this study are the utilization of various types and amounts of PA and sedentary behavior using the validated questionnaire collected by trained personnel with a systematic protocol, the prospective cohort design with a follow-up over 8 years for all-cause and cause-specific mortality, a variety of clinical and metabolic variables, and a large number of recent multiethnic cohorts (2007–2014) that represent the current US population. Therefore, we believe our findings are generalizable to the US population. However, several limitations must be noted. First, we defined NAFLD using non-invasive panels (in the absence of any steatogenic medication(s) and other known causes of chronic liver disease), which may misclassify, overestimate, and/or underestimate the true prevalence of NAFLD and may limit the accuracy of identifying NAFLD in the general population. The NHANES 2007–2014 lacks radiological and histological data, considered the gold standard for diagnosing NAFLD. The HSI and USFLI, on the other hand, have been validated as robust non-invasive panels for the detecting NAFLD and independent predictors of all-cause and liver-related mortality [14,15,18,19,26]. Second, the NHANES data does not provide the longitudinal PA questionnaire, clinical, or laboratory data. Therefore, we are unable to assess serial changes in NAFLD status, PA status, weight, or metabolic changes related to PA in this study. Third, we assessed the types of PA status from a self-reported questionnaire rather than objectively measured PA, although this PA questionnaire was based on the well-validated Global Physical Activity Questionnaire. Fourth, we were unable to analyze and report liver-related mortality, which was not publicly available due to the small number of deaths. In addition, the data describing the severity of liver disease and the occurrence of liver outcomes were not included in the NHANES study design. Finally, unmeasured residual confounders must be considered as a possible explanation for at least part of the association, although we tried to adjust known risk factors to determine the independent association between the types of PA and mortality.

In conclusion, this population-based study suggests that meeting the PA guidelines of >150 min/week for leisure-time and transportation-related PA may provide a survival benefit for all-cause and cardiovascular mortality among individuals with NAFLD. Sedentary behavior in the setting of NAFLD demonstrated a survival disadvantage in all-cause and cardiovascular mortality. Therefore, we suggest the implementation of increasing PA and decreasing sedentary behavior in the management plan for the rapidly rising population of individuals with NAFLD. In the future, these lifestyle modifications can be easily integrated with approved pharmacologic therapy.

## Figures and Tables

**Table 1:** Characteristics of Study Participants based on Total Physical Activity Status (*n* = 10,853).

	Physically Inactive	Physically Active	*p*-Value
Age (years)	51.8 ± 0.3	45.1 ± 0.3	<0.001
Sex (Men)	37.0 (35.2–38.9)	56.2 (54.5–57.8)	<0.001
Body mass index (kg/m^2^)	34.1 ± 0.1	32.6 ± 0.1	<0.001
Waist circumference (cm)	107.8 ± 0.5	106.1 ± 0.3	0.002
Hypertension (%)	46.2 (44.2–48.2)	31.2 (29.6–32.9)	<0.001
Diabetes (%)	22.6 (21.3–24.0)	11.9 (10.9–12.9)	<0.001
Ethnicity (%)			0.559
Non-Hispanic white	65.7 (61.4–69.9)	66.4 (62.1–70.5)	
Non-Hispanic black	12.5 (10.5–14.9)	11.5 (9.7–13.6)	
Hispanics	16.7 (13.6–20.4)	17.0 (14.1–20.3)	
Others	5.0 (4.2–6.1)	5.1 (4.3–6.1)	
Smoking (%)			0.485
Never	55.7 (53.8–57.6)	56.9 (54.8–58.9)	
Current smoker	17.3 (16.0–18.8)	17.6 (16.4–18.8)	
Ex-smoker	26.9 (25.2–28.8)	25.6 (23.9–27.3)	
High education (%)	77.4 (75.2–79.5)	83.9 (82.1–85.5)	<0.001
Married status (%)	64.7 (62.9–66.4)	66.3 (64.7–67.9)	0.102
Poverty (%)	16.6 (14.8–18.6)	14.1 (12.6–15.6)	0.003
Total cholesterol (mg/dL)	195.6 ± 0.7	196.9 ± 0.7	0.147
Fasting Triglyceride (mg/dL)	150.8 ± 3.8	148.4 ± 2.9	0.558
High-density lipoprotein-cholesterol (mg/dL)	48.3 ± 0.3	47.5 ± 0.2	0.007
Fasting glucose (mg/dL)	115.9 ± 1.3	108.5 ± 0.7	<0.001
Hemoglobin A1c (%)	5.9 ± 0.02	5.7 ± 0.01	<0.001
Fasting insulin (pmol/L)	115.3 ± 3.0	98.3 ± 2.2	0.008
Alanine aminotransferase (IU/L)	27.6 ± 0.4	30.2 ± 0.3	<0.001
Aspartate aminotransferase (IU/L)	25.4 ± 0.2	26.0 ± 0.2	0.028
Gamma-glutamyl transferase (IU/L)	30.7 ± 0.6	30.2 ± 0.5	0.565

Data are expressed as the weighted mean ± standard error or weighted frequency (95% confidence intervals).We categorized physical activity as physically inactive, who did not meet guidelines, and physically active, who met guidelines according to the 2018 Physical Activity Guidelines for Americans (adults engage in ≥150 min/week of moderate-intensity activity per week, 75 min/week of vigorous-intensity activity per week, or an equivalent combination).Fasting triglyceride (*n* = 5306), fasting glucose (*n* = 5301), and fasting insulin (*n* = 5241) were analyzed in fasting individuals only.

**Table 2:** Multivariable Hazard Ratio for All-Cause Mortality based on the Meeting Physical Activity Guidelines among Individuals with NAFLD as defined by the Hepatic Steatosis Index.

	Age, Sex-Adjusted Model		Multivariable Model 1		Multivariable Model 2	
	HR (95% CI)	*p*-Value	HR (95% CI)	*p*-Value	HR (95% CI)	*p*-Value
Total physical activity						
<150 (min/week)	1		1		1	
≥150 (min/week)	0.63 (0.52–0.75)	<0.001	0.74 (0.60–0.90)	0.003	0.75 (0.62–0.92)	0.006
Leisure-time physical activity						
<150 (min/week)	1		1		1	
≥150 (min/week)	0.66 (0.52–0.84)	0.001	0.76 (0.59–0.98)	0.035	0.76 (0.59–0.98)	0.033
Occupation-related physical activity						
<150 (min/week)	1		1		1	
≥150 (min/week)	0.78 (0.64–0.95)	0.013	0.90 (0.73–1.11)	0.303	0.92 (0.74–1.14)	0.446
Transportation-related physical activity						
<150 (min/week)	1		1		1	
≥150 (min/week)	0.62 (0.46–0.85)	0.004	0.62 (0.45–0.86)	0.005	0.63 (0.45–0.88)	0.007

The multivariable model 1 was adjusted for age, sex, race/ethnicity, education level, married status, economic status, smoking status, hypertension, diabetes, total cholesterol, total calorie intake, caffeine consumption, and alcohol consumption using appropriate sampling weights. The multivariable model 2 was adjusted for waist circumference in addition to model 1 using appropriate sampling weights. Abbreviations: NAFLD, nonalcoholic fatty liver disease; HR, hazard ratio; CI, confidence interval.

**Table 3:** Multivariable Hazard Ratio for All-Cause Mortality based on the Amount of Physical Activity among Individuals with NAFLD as defined by the Hepatic Steatosis Index.

	Age, Sex-Adjusted Model		Multivariable Model 1		Multivariable Model 2	
	HR (95% CI)	*p*-Value	HR (95% CI)	*p*-Value	HR (95% CI)	*p*-Value
Total physical activity (min/week)						
0	1	<0.001 *	1	0.001 *	1	0.002 *
1–149	0.57 (0.47–0.70)	<0.001	0.59 (0.47–0.75)	<0.001	0.61 (0.48–0.78)	<0.001
150–299	0.51 (0.37–0.69)	<0.001	0.59 (0.42–0.84)	0.004	0.59 (0.42–0.84)	0.004
300–	0.56 (0.46–0.68)	<0.001	0.67 (0.54–0.82)	<0.001	0.68 (0.55–0.85)	0.001
Leisure-time physical activity (min/week)						
0	1	<0.001 *	1	0.006 *	1	0.008 *
1–149	0.64 (0.52–0.80)	<0.001	0.71 (0.55–0.90)	0.006	0.73 (0.57–0.94)	0.015
150–299	0.61 (0.45–0.84)	0.003	0.68 (0.48–0.97)	0.034	0.67 (0.47–0.97)	0.033
300–	0.61 (0.46–0.80)	0.001	0.74 (0.56–0.98)	0.034	0.75 (0.56–0.996)	0.047
Occupation-related physical activity (min/week)						
0	1	0.004 *	1	0.155 *	1	0.273 *
1–149	0.56 (0.40–0.78)	0.001	0.55 (0.39–0.76)	0.001	0.57 (0.41–0.79)	0.001
150–299	0.55 (0.36–0.82)	0.005	0.62 (0.39–0.98)	0.041	0.63 (0.40–1.00)	0.050
300–	0.78 (0.64–0.95)	0.015	0.89 (0.72–1.10)	0.286	0.92 (0.74–1.15)	0.449
Transportation-related physical activity (min/week)						
0	1	0.001 *	1	0.002 *	1	0.004 *
1–149	0.77 (0.57–1.02)	0.072	0.81 (0.59–1.09)	0.162	0.81 (0.59–1.11)	0.187
150–299	0.65 (0.39–1.07)	0.088	0.58 (0.31–1.06)	0.076	0.59 (0.32–1.08)	0.088
300–	0.59 (0.40–0.87)	0.008	0.62 (0.42–0.92)	0.020	0.63 (0.42–0.94)	0.026

The multivariable model 1 was adjusted for age, sex, race/ethnicity, education level, marital status, economic status, smoking status, hypertension, diabetes, total cholesterol, total calorie intake, caffeine consumption, and alcohol consumption using appropriate sampling weights. The multivariable model 2 was adjusted for waist circumference in addition to model 1 using appropriate sampling weights. Abbreviations: NAFLD, nonalcoholic fatty liver disease; HR, hazard ratio; CI, confidence interval. * *p*-values were analyzed using the test of the trend of hazards.

**Table 4:** Multivariable Hazard Ratio for Cardiovascular Mortality based on the Meeting Physical Activity Guideline among Individuals with NAFLD as defined by the Hepatic Steatosis Index.

	Age, Sex-Adjusted Model		Multivariable Model 1		Multivariable Model 2	
	HR (95% CI)	*p*-Value	HR (95% CI)	*p*-Value	HR (95% CI)	*p*-Value
Total physical activity						
<150 (min/week)	1		1		1	
≥150 (min/week)	0.55 (0.42–0.71)	<0.001	0.65 (0.49–0.87)	0.005	0.66 (0.50–0.89)	0.006
Leisure-time physical activity						
<150 (min/week)	1		1		1	
≥150 (min/week)	0.56 (0.40–0.80)	0.002	0.63 (0.44–0.91)	0.015	0.61 (0.42–0.88)	0.009
Occupation-related physical activity						
<150 (min/week)	1		1		1	
≥150 (min/week)	0.75 (0.54–1.04)	0.081	0.90 (0.63–1.28)	0.552	0.95 (0.66–1.36)	0.785
Transportation-related physical activity						
<150 (min/week)	1		1		1	
≥150 (min/week)	0.38 (0.24–0.62)	<0.001	0.38 (0.23–0.65)	0.001	0.40 (0.23–0.68)	0.001

The multivariable model 1 was adjusted for age, sex, race/ethnicity, education level, marital status, economic status, smoking status, hypertension, diabetes, total cholesterol, total calorie intake, caffeine consumption, and alcohol consumption using appropriate sampling weights. The multivariable model 2 was adjusted for waist circumference in addition to model 1 using appropriate sampling weights. Abbreviations: NAFLD, nonalcoholic fatty liver disease; HR, hazard ratio; CI, confidence interval.

**Table 5:** Multivariable Hazard Ratio for Cardiovascular Mortality based on the Amount of Physical Activity among Individuals with NAFLD as defined by the Hepatic Steatosis Index.

	Age, Sex-Adjusted Model		Multivariable Model 1		Multivariable Model 2	
	HR (95% CI)	*p*-Value	HR (95% CI)	*p*-Value	HR (95% CI)	*p*-Value
Total physical activity (min/week)						
0	1	<0.001 *	1	0.001 *	1	0.002 *
1–149	0.51 (0.37–0.70)	<0.001	0.57 (0.40–0.80)	0.002	0.62 (0.43–0.88)	0.009
150–299	0.62 (0.40–0.94)	0.027	0.75 (0.47–1.19)	0.219	0.73 (0.46–1.17)	0.190
300–	0.42 (0.30–0.57)	<0.001	0.51 (0.36–0.73)	<0.001	0.54 (0.38–0.78)	0.001
Leisure-time physical activity (min/week)						
0	1	<0.001 *	1	<0.001 *	1	<0.001 *
1–149	0.69 (0.48–1.00)	0.052	0.80 (0.54–1.20)	0.281	0.85 (0.57–1.27)	0.417
150–299	0.78 (0.52–1.16)	0.214	0.86 (0.56–1.32)	0.496	0.83 (0.54–1.26)	0.379
300–	0.29 (0.18–0.46)	<0.001	0.34 (0.21–0.55)	<0.001	0.35 (0.21–0.58)	<0.001
Occupation-related physical activity (min/week)						
0	1	0.020 *	1	0.238 *	1	0.397 *
1–149	0.34 (0.16–0.72)	0.006	0.38 (0.18–0.83)	0.001	0.41 (0.19–0.86)	0.020
150–299	0.75 (0.38–1.46)	0.390	0.97 (0.49–1.93)	0.041	1.03 (0.52–2.05)	0.937
300–	0.67 (0.47–0.96)	0.029	0.80 (0.54–1.18)	0.252	0.85 (0.57–1.25)	0.402
Transportation-related physical activity (min/week)						
0	1	<0.001 *	1	0.001 *	1	0.001 *
1–149	0.83 (0.53–1.29)	0.401	0.86 (0.53–1.39)	0.527	0.92 (0.57–1.49)	0.736
150–299	0.52 (0.24–1.15)	0.107	0.53 (0.21–1.32)	0.171	0.55 (0.21–1.39)	0.201
300–	0.28 (0.15–0.51)	<0.001	0.28 (0.14–0.55)	<0.001	0.30 (0.15–0.58)	0.001

The multivariable model 1 was adjusted for age, sex, race/ethnicity, education level, marital status, economic status, smoking status, hypertension, diabetes, total cholesterol, total calorie intake, caffeine consumption, and alcohol consumption using appropriate sampling weights. The multivariable model 2 was adjusted for waist circumference in addition to model 1 using appropriate sampling weights. Abbreviations: NAFLD, nonalcoholic fatty liver disease; HR, hazard ratio; CI, confidence interval. * *p*-values were analyzed using the test of the trend of hazards.

**Table 6:** Multivariable Hazard Ratio for All-Cause and Cardiovascular Mortality based on the Amount of Sitting Time among Individuals with NAFLD as defined by the Hepatic Steatosis Index.

	Age, Sex-Adjusted Model		Multivariable Model 1		Multivariable Model 2	
	OR (95% CI)	*p*-Value	OR (95% CI)	*p*-Value	OR (95% CI)	*p*-Value
All-cause mortality						
Sitting time						
Q1 (<4 h)	1	<0.001 *	1	<0.001 *	1	0.002 *
Q2 (4–<6 h)	0.96 (0.77–1.21)	0.751	0.95 (0.74–1.21)	0.670	0.95 (0.75–1.20)	0.648
Q3 (6–<8 h)	1.12 (0.87–1.45)	0.350	1.03 (0.80–1.34)	0.800	1.05 (0.82–1.37)	0.674
Q4 (≥8 h)	1.46 (1.23–1.73)	<0.001	1.32 (1.10–1.57)	0.003	1.27 (1.06–1.53)	0.010
Total physical activity (min/week)						
0			1	0.007 *	1	0.013 *
1–149			0.61 (0.48–0.78)	<0.001	0.63 (0.49–0.80)	<0.001
150–299			0.62 (0.44–0.88)	0.008	0.62 (0.43–0.88)	0.009
300–			0.73 (0.59–0.90)	0.004	0.75 (0.60–0.92)	0.008
Cardiovascular mortality						
Sitting time						
Q1 (<4 h)	1	<0.001 *	1	<0.001 *	1	0.003 *
Q2 (4–<6 h)	1.04 (0.71–1.53)	0.839	1.11 (0.71–1.71)	0.646	1.10 (0.71–1.71)	0.663
Q3 (6–<8 h)	1.28 (0.82–2.02)	0.276	1.40 (0.85–2.31)	0.178	1.43 (0.87–2.38)	0.159
Q4 (≥8 h)	1.93 (1.36–2.74)	<0.001	1.79 (1.23–2.62)	0.003	1.62 (1.10–2.39)	0.015
Total physical activity (min/week)						
0			1	0.005 *	1	0.009 *
1–149			0.58 (0.41–0.83)	0.003	0.63 (0.44–0.90)	0.013
150–299			0.78 (0.49–1.25)	0.305	0.76 (0.47–1.21)	0.249
300–			0.59 (0.41–0.83)	0.003	0.60 (0.42–0.86)	0.006

The multivariable model 1 was adjusted for age, sex, race/ethnicity, education level, marital status, economic status, smoking status, hypertension, diabetes, total cholesterol, total physical activity, total calorie intake, caffeine consumption, and alcohol consumption using appropriate sampling weights. The multivariable model 2 was adjusted for waist circumference in addition to model 1 using appropriate sampling weights. Abbreviations: NAFLD, nonalcoholic fatty liver disease; OR, odds ratio; CI, confidence interval. Weighted quartile 1, <4 h; quartile 2, ≥4 h to <6 h; quartile 3, ≥6 h to <8 h; quartile 4, ≥8 h. * *p*-values were analyzed using the test of the trend of hazards.

## Data Availability

The National Health and Nutrition Examination Survey 2007–2014 datasets are publicly available at the National Center for Health Statistics of the Center for Disease Control and Prevention (https://www.cdc.gov/nchs/nhanes/index.htm, accessed on 1 July 2022).

## Supplementary Materials

The following supporting information can be downloaded at: https://www.mdpi.com/article/10.3390/jcm12051923/s1. Table S1: Multivariable Hazard Ratio for All-Cause Mortality based on the Meeting Physical Activity Guideline among Individuals with NAFLD as defined by the US Fatty Liver Index; Table S2: Multivariable Hazard Ratio for All-Cause Mortality based on the Amount of Physical Activity among Individuals with NAFLD as defined by the US Fatty Liver Index; Table S3: Multivariable Hazards Ratio for All-Cause Mortality based on the Meeting Physical Activity Guidelines among Individuals with NAFLD defined by Hepatic Steatosis Index.; Table S4: Multivariable Hazards Ratio for All-Cause Mortality based on the Meeting Physical Activity Guidelines among Individuals with NAFLD defined by Hepatic Steatosis Index; Table S5: Multivariable Hazards Ratio for All-Cause Mortality based on the Meeting Physical Activity Guidelines among Individuals with Advanced Fibrosis; Table S6: Multivariable Hazards Ratio for Cardiovascular Mortality based on the Meeting Physical Activity Guideline among Individuals with NAFLD defined by US Fatty Liver Index. Table S7: Multivariable Hazards Ratio for Cardiovascular Mortality based on the Amount of Physical Activity among Individuals with NAFLD defined by US Fatty Liver Index. Table S8: Multivariable Hazards Ratio for All-Cause Mortality based on the Meeting Physical Activity Guidelines among Individuals without NAFLD defined by Hepatic Steatosis Index. Table S9: Multivariable Hazards Ratio for All-Cause Mortality based on the Amount of Physical Activity among Individuals without NAFLD defined by Hepatic Steatosis Index. Table S10: Multivariable Hazards Ratio for Cardiovascular Mortality based on the Meeting Physical Activity Guideline among Individuals without NAFLD defined by Hepatic Steatosis Index. Table S11: Multivariable Hazards Ratio for Cancer-related Mortality based on the Meeting Physical Activity Guideline among Individuals with NAFLD defined by Hepatic Steatosis Index. Table S12: Multivariable Hazards Ratio for Cancer-related Mortality based on the Meeting Physical Activity Guideline among Individuals without NAFLD defined by Hepatic Steatosis Index. Table S13: Multivariable Hazards Ratio for All-Cause and Cardiovascular Mortality based on the Amount of Sitting Time among Individuals with NAFLD defined by US Fatty Liver Index. Table S14: Multivariable Hazards Ratio for All-Cause and Cardiovascular Mortality based on the Amount of Sitting Time among Individuals without NAFLD defined by Hepatic Steatosis Index.

## Abbreviations

BMI: body mass index; CI, confidence interval; HR, hazard ratio; HSI, hepatic steatosis index; NAFLD, nonalcoholic fatty liver disease; NHANES, the National Health and Nutrition Examination Survey; PA, physical activity; UCOD_113, underlying cause of death 113; USFLI, US fatty liver index.
