# Supplementary material for: Types of Physical Activity in Nonalcoholic Fatty Liver Disease and All-Cause and Cardiovascular Mortality
Source: J Clin Med. 2023 Feb 28;12(5):1923. doi: 10.3390/jcm12051923 (PMC10004264; doi:10.3390/jcm12051923)
Supplement: Supplementary file 1 [file jcm-12-01923-s001.zip › jcm-2223049-supplementary.pdf]

Supplementary Materials

Table S1. Multivariable Hazards Ratio for All-Cause Mortality based on the Meeting Physical Activity Guideline among Individuals with NAFLD defined by US Fatty Liver Index.

	Age, sex-adjusted model		Multivariable model 1		Multivariable model 2	
	HR (95% CI)	*p*-value	HR (95% CI)	*p*-value	HR (95% CI)	*p*-value
Total physical activity						
<150 (min/wk)	1		1		1	
≥150 (min/wk)	0.63 (0.47–0.84)	0.002	0.70 (0.52–0.95)	0.025	0.72 (0.53–0.97)	0.034
Leisure-time physical activity						
<150 (min/wk)	1		1		1	
≥150 (min/wk)	0.45 (0.31–0.63)	<0.001	0.48 (0.33–0.68)	<0.001	0.48 (0.34–0.69)	<0.001
Occupation-related physical activity						
<150 (min/wk)	1		1		1	
≥150 (min/wk)	0.86 (0.62–1.19)	0.358	0.95 (0.69–1.32)	0.773	0.97 (0.70–1.36)	0.863
Transportation-related physical activity						
<150 (min/wk)	1		1		1	
≥150 (min/wk)	0.65 (0.42–1.04)	0.071	0.68 (0.40–1.15)	0.148	0.70 (0.41–1.17)	0.167

The multivariable model 1 was adjusted for age, sex, race/ethnicity, education level, marital status, economic status, smoking status, hypertension, diabetes, total cholesterol, total calorie intake, caffeine consumption, and alcohol consumption using appropriate sampling weights. The multivariable model 2 was adjusted for waist circumference in addition to model 1 using appropriate sampling weights. Abbreviations: NAFLD, nonalcoholic fatty liver disease; HR, hazard ratio; CI, confidence interval.

Table S2. Multivariable Hazards Ratio for All-Cause Mortality based on the Amount of Physical Activity among Individuals with NAFLD defined by US Fatty Liver Index.

	Age, sex-adjusted model		Multivariable model 1		Multivariable model 2	
	HR (95% CI)	*p*-value	HR (95% CI)	*p*-value	HR (95% CI)	*p*-value
Total physical activity (min/wk)						
0	1	<0.001 *	1	0.006 *	1	0.009 *
1–149	0.56 (0.41–0.77)	<0.001	0.64 (0.46–0.90)	0.010	0.66 (0.47–0.90)	0.011
150–299	0.50 (0.29–0.87)	0.015	0.58 (0.33–1.03)	0.061	0.59 (0.34–1.04)	0.066
300-	0.56 (0.42–0.74)	<0.001	0.64 (0.47–0.86)	0.003	0.64 (0.49–0.88)	0.006
Leisure-time physical activity (min/wk)						
0	1	<0.001 *	1	<0.001 *	1	<0.001 *
1–149	0.54 (0.37–0.81)	0.003	0.65 (0.43–0.99)	0.045	0.65 (0.44–1.01)	0.055
150–299	0.36 (0.20–0.65)	0.001	0.37 (0.21–0.70)	0.002	0.39 (0.21–0.70)	0.002
300-	0.45 (0.33–0.63)	<0.001	0.51 (0.36–0.72)	<0.001	0.52 (0.36–0.73)	<0.001
Occupation-related physical activity (min/wk)						
0	1	0.204 *	1	0.572 *	1	0.655 *
1–149	0.70 (0.41–1.17)	0.171	0.80 (0.48–1.33)	0.376	0.79 (0.46–1.33)	0.365
150–299	0.84 (0.44–1.61)	0.590	1.01 (0.50–2.03)	0.986	1.03 (0.51–2.06)	0.943
300-	0.83 (0.61–1.13)	0.228	0.92 (0.68–1.23)	0.555	0.93 (0.69–1.26)	0.641
Transportation-related physical activity (min/wk)						
0	1	0.058 *	1	0.155 *	1	0.177 *
1–149	0.76 (0.47–1.22)	0.252	0.85 (0.51–1.40)	0.512	0.86 (0.52–1.41)	0.542
150–299	0.71 (0.40–1.28)	0.256	0.72 (0.38–1.34)	0.296	0.73 (0.40–1.35)	0.312
300-	0.60 (0.27–1.31)	0.192	0.63 (0.26–1.51)	0.295	0.65 (0.27–1.56)	0.326

The multivariable model 1 was adjusted for age, sex, race/ethnicity, education level, marital status, economic status, smoking status, hypertension, diabetes, total cholesterol, total calorie intake, caffeine consumption, and alcohol consumption using appropriate sampling weights. The multivariable model 2 was adjusted for waist circumference in addition to model 1 using appropriate sampling weights. Abbreviations: NAFLD, nonalcoholic fatty liver disease; HR, hazard ratio; CI, confidence interval. * *p*-values were analyzed using the test of the trend of hazards.

Table S3. Multivariable Hazards Ratio for All-Cause Mortality based on the Meeting Physical Activity Guidelines among Individuals with NAFLD defined by Hepatic Steatosis Index.

	Age, sex-adjusted model		Multivariable model 1		Multivariable model 2	
	**HR (95% CI)**	***p*-value**	**HR (95% CI)**	***p*-value**	**HR (95% CI)**	***p*-value**
Total physical activity						
<150 (min/wk)	1		1		1	
≥150 (min/wk)	0.63 (0.53–0.76)	<0.001	0.75 (0.61–0.91)	0.005	0.76 (0.62–0.93)	0.008
Obese NAFLD	1.28 (1.09–1.51)	0.004	1.19 (1.00–1.42)	0.052	1.22 (1.01–1.48)	0.044
Total physical activity						
Total physical activity						
<150 (min/wk)	1		1		1	
≥150 (min/wk)	0.66 (0.55–0.80)	<0.001	0.74 (0.60–0.90)	0.003	0.75 (0.62–0.92)	0.006
Diabetic NAFLD	1.59 (1.36–1.86)	<0.001	1.50 (1.25–1.80)	<0.001	1.54 (1.26–1.89)	<0.001

The multivariable model 1 was adjusted for age, sex, race/ethnicity, education level, married status, economic status, smoking status, hypertension, diabetes, total cholesterol, total calorie intake, caffeine consumption, and alcohol consumption using appropriate sampling weights. The multivariable model 2 was adjusted for waist circumference in addition to model 1 using appropriate sampling weights. Abbreviations: NAFLD, nonalcoholic fatty liver disease; HR, hazard ratio; CI, confidence interval.

Table S4. Multivariable Hazards Ratio for All-Cause Mortality based on the Meeting Physical Activity Guidelines among Individuals with NAFLD defined by Hepatic Steatosis Index.

	Age, sex-adjusted model		Multivariable model 1		Multivariable model 2	
	**HR (95% CI)**	***p*-value**	**HR (95% CI)**	***p*-value**	**HR (95% CI)**	***p*-value**
Total physical activity						
<150 (min/wk)	1		1		1	
≥150 (min/wk)	0.63 (0.53–0.76)	<0.001	0.74 (0.61–0.91)	0.004	0.75 (0.62–0.92)	0.006
Advanced fibrosis	1.63 (1.35–1.97)	<0.001	1.51 (1.21–1.89)	<0.001	1.47 (1.14–1.88)	0.003
Leisure-time physical activity						
<150 (min/wk)	1		1		1	
≥150 (min/wk)	0.66 (0.52–0.85)	0.001	0.77 (0.59–0.99)	0.045	0.76 (0.59–0.99)	0.039
Advanced fibrosis	1.64 (1.36–1.99)	<0.001	1.51 (1.21–1.89)	<0.001	1.46 (1.14–1.88)	0.003
Occupation-related physical activity						
<150 (min/wk)	1		1		1	
≥150 (min/wk)	0.79 (0.65–0.96)	0.018	0.90 (0.73–1.11)	0.342	0.93 (0.75–1.15)	0.480
Advanced fibrosis	1.64 (1.36–1.99)	<0.001	1.52 (1.22–1.90)	<0.001	1.47 (1.15–1.88)	0.003
Transportation-related physical activity						
<150 (min/wk)	1		1		1	
≥150 (min/wk)	0.63 (0.46–0.86)	0.005	0.62 (0.44–0.86)	0.005	0.63 (0.45–0.88)	0.007
Advanced fibrosis	1.66 (1.37–2.00)	<0.001	1.53 (1.22–1.90)	<0.001	1.47 (1.15–1.89)	0.003

The multivariable model 1 was adjusted for age, sex, race/ethnicity, education level, married status, economic status, smoking status, hypertension, diabetes, total cholesterol, total calorie intake, caffeine consumption, and alcohol consumption using appropriate sampling weights. The multivariable model 2 was adjusted for waist circumference in addition to model 1 using appropriate sampling weights. Abbreviations: NAFLD, nonalcoholic fatty liver disease; HR, hazard ratio; CI, confidence interval.

Table S5. Multivariable Hazards Ratio for All-Cause Mortality based on the Meeting Physical Activity Guidelines among Individuals with Advanced Fibrosis.

	**Age, sex-adjusted model**		**Multivariable model 1**		**Multivariable model 2**	
	**HR (95% CI)**	***p*-value**	**HR (95% CI)**	***p*-value**	**HR (95% CI)**	***p*-value**
Total physical activity						
<150 (min/wk)	1		1		1	
≥150 (min/wk)	0.43 (0.31–0.58)	<0.001	0.52 (0.38–0.71)	<0.001	0.53 (0.37–0.73)	<0.001
Leisure-time physical activity						
<150 (min/wk)	1		1		1	
≥150 (min/wk)	0.51 (0.38–0.70)	<0.001	0.65 (0.47–0.90)	0.009	0.63 (0.45–0.88)	0.007
Occupation-related physical activity						
<150 (min/wk)	1		1		1	
≥150 (min/wk)	0.53 (0.34–0.82)	0.005	0.64 (0.40–1.01)	0.054	0.66 (0.42–1.04)	0.013
Transportation-related physical activity						
<150 (min/wk)	1		1		1	
≥150 (min/wk)	0.46 (0.26–0.81)	0.008	0.45 (0.24–0.84)	0.013	0.45 (0.24–0.85)	0.014

The multivariable model 1 was adjusted for age, sex, race/ethnicity, education level, married status, economic status, smoking status, hypertension, diabetes, total cholesterol, total calorie intake, caffeine consumption, and alcohol consumption using appropriate sampling weights. The multivariable model 2 was adjusted for waist circumference in addition to model 1 using appropriate sampling weights. Abbreviations: NAFLD, nonalcoholic fatty liver disease; HR, hazard ratio; CI, confidence interval.

Table S6. Multivariable Hazards Ratio for Cardiovascular Mortality based on the Meeting Physical Activity Guideline among Individuals with NAFLD defined by US Fatty Liver Index.

	**Age, sex-adjusted model**		**Multivariable model 1**		**Multivariable model 2**	
	**HR (95% CI)**	***p*-value**	**HR (95% CI)**	***p*-value**	**HR (95% CI)**	***p*-value**
Total physical activity						
<150 (min/wk)	1		1		1	
≥150 (min/wk)	0.51 (0.30–0.84)	0.009	0.53 (0.32–0.91)	0.021	0.55 (0.32–0.97)	0.038
Leisure-time physical activity						
<150 (min/wk)	1		1		1	
≥150 (min/wk)	0.36 (0.22–0.61)	<0.001	0.36 (0.20–0.63)	0.001	0.37 (0.21–0.65)	0.001
Occupation-related physical activity						
<150 (min/wk)	1		1		1	
≥150 (min/wk)	0.87 (0.51–1.48)	0.594	0.94 (0.54–1.62)	0.812	0.97 (0.55–1.70)	0.914
Transportation-related physical activity						
<150 (min/wk)	1		1		1	
≥150 (min/wk)	0.35 (0.15–0.81)	0.015	0.30 (0.12–0.73)	0.009	0.31 (0.13–0.77)	0.012

The multivariable model 1 was adjusted for age, sex, race/ethnicity, education level, marital status, economic status, smoking status, hypertension, diabetes, total cholesterol, total calorie intake, caffeine consumption, and alcohol consumption using appropriate sampling weights. The multivariable model 2 was adjusted for waist circumference in addition to model 1 using appropriate sampling weights.Abbreviations: NAFLD, nonalcoholic fatty liver disease; HR, hazard ratio; CI, confidence interval.

Table S7. Multivariable Hazards Ratio for Cardiovascular Mortality based on the Amount of Physical Activity among Individuals with NAFLD defined by US Fatty Liver Index.

	Age, sex-adjusted model		Multivariable model 1		Multivariable model 2	
	HR (95% CI)	*p*-value	HR (95% CI)	*p*-value	HR (95% CI)	*p*-value
Total physical activity (min/wk)						
0	1	0.005 *	1	0.016 *	1	0.030 *
1–149	0.66 (0.24–1.26)	0.206	0.72 (0.35–1.47)	0.364	0.75 (0.37–1.51)	0.410
150–299	0.46 (0.20–1.08)	0.075	0.50 (0.20–1.28)	0.146	0.52 (0.20–1.37)	0.183
300-	0.45 (0.26–0.78)	0.005	0.48 (0.26–0.88)	0.018	0.50 (0.27–0.94)	0.032
Leisure-time physical activity (min/wk)						
0	1	<0.001 *	1	0.002 *	1	0.002 *
1–149	0.89 (0.47–1.66)	0.707	1.06 (0.53–2.14)	0.868	1.11 (0.55–2.22)	0.768
150–299	0.49 (0.26–0.92)	0.027	0.48 (0.24–0.95)	0.035	0.49 (0.24–0.96)	0.038
300-	0.23 (0.10–0.54)	0.001	0.24 (0.09–0.64)	0.005	0.25 (0.09–0.67)	0.007
Occupation-related physical activity (min/wk)						
0	1	0.389 *	1	0.587 *	1	0.672 *
1–149	0.43 (0.11–1.70)	0.223	0.47 (0.12–1.85)	0.273	0.46 (0.12–1.81)	0.250
150–299	0.80 (0.27–2.34)	0.673	0.94 (0.31–2.84)	0.911	0.97 (0.32–2.92)	0.960
300-	0.81 (0.47–1.39)	0.446	0.86 (0.49–1.53)	0.614	0.89 (0.50–1.60)	0.701
Transportation-related physical activity (min/wk)						
0	1	0.012 *	1	0.010 *	1	0.013 *
1–149	0.88 (0.45–1.74)	0.714	0.91 (0.46–1.82)	0.795	0.95 (0.48–1.84)	0.868
150–299	0.52 (0.18–1.50)	0.219	0.40 (0.12–1.28)	0.120	0.41 (0.12–1.37)	0.144
300-	0.21 (0.04–1.00)	0.050	0.22 (0.04–1.06)	0.058	0.23 (0.05–1.11)	0.066

The multivariable model 1 was adjusted for age, sex, race/ethnicity, education level, marital status, economic status, smoking status, hypertension, diabetes, total cholesterol, total calorie intake, caffeine consumption, and alcohol consumption using appropriate sampling weights. The multivariable model 2 was adjusted for waist circumference in addition to model 1 using appropriate sampling weights. Abbreviations: NAFLD, nonalcoholic fatty liver disease; HR, hazard ratio; CI, confidence interval. * *p*-values were analyzed using the test of the trend of hazards.

Table S8. Multivariable Hazards Ratio for All-Cause Mortality based on the Meeting Physical Activity Guidelines among Individuals without NAFLD defined by Hepatic Steatosis Index.

	Age, sex-adjusted model		Multivariable model 1		Multivariable model 2	
	HR (95% CI)	*p*-value	HR (95% CI)	*p*-value	HR (95% CI)	*p*-value
Total physical activity						
<150 (min/wk)	1		1		1	
≥150 (min/wk)	0.43 (0.36–0.50)	<0.001	0.48 (0.40–0.56)	<0.001	0.50 (0.42–0.60)	<0.001
Leisure-time physical activity						
<150 (min/wk)	1		1		1	
≥150 (min/wk)	0.40 (0.32–0.48)	<0.001	0.46 (0.37–0.56)	<0.001	0.47 (0.38–0.59)	<0.001
Occupation-related physical activity						
<150 (min/wk)	1		1		1	
≥150 (min/wk)	0.70 (0.60–0.83)	0.013	0.75 (0.63–0.90)	0.002	0.79 (0.66–0.95)	0.013
Transportation-related physical activity						
<150 (min/wk)	1		1		1	
≥150 (min/wk)	0.71 (0.53–0.94)	0.018	0.75 (0.56–1.00)	0.050	0.77 (0.58–1.03)	0.077

The multivariable model 1 was adjusted for age, sex, race/ethnicity, education level, married status, economic status, smoking status, hypertension, diabetes, total cholesterol, total calorie intake, caffeine consumption, and alcohol consumption using appropriate sampling weights. The

multivariable model 2 was adjusted for waist circumference in addition to model 1 using appropriate sampling weights. Abbreviations: NAFLD, nonalcoholic fatty liver disease; HR, hazard ratio; CI, confidence interval.

Table S9. Multivariable Hazards Ratio for All-Cause Mortality based on the Amount of Physical Activity among Individuals without NAFLD defined by Hepatic Steatosis Index.

	**Age, sex-adjusted model**		**Multivariable model 1**		**Multivariable model 2**	
	**HR (95% CI)**	***p*-value**	**HR (95% CI)**	***p*-value**	**HR (95% CI)**	***p*-value**
Total physical activity (min/wk)						
0	1	<0.001 *	1	<0.001 *	1	<0.001 *
1–149	0.65 (0.53–0.80)	<0.001	0.73 (0.57–0.94)	0.016	0.76 (0.59–0.98)	0.033
150–299	0.34 (0.27–0.44)	<0.001	0.41 (0.32–0.51)	<0.001	0.43 (0.34–0.55)	<0.001
300-	0.38 (0.31–0.46)	<0.001	0.43 (0.35–0.54)	<0.001	0.46 (0.37–0.58)	<0.001
Leisure-time physical activity (min/wk)						
0	1	<0.001 *	1	<0.001 *	1	<0.001 *
1–149	0.53 (0.44–0.65)	<0.001	0.61 (0.49–0.77)	<0.001	0.64 (0.51–0.80)	<0.001
150–299	0.35 (0.26–0.49)	<0.001	0.42 (0.31–0.59)	<0.001	0.44 (0.32–0.61)	<0.001
300-	0.34 (0.27–0.43)	<0.001	0.40 (0.30–0.53)	<0.001	0.41 (0.31–0.55)	<0.001
Occupation-related physical activity (min/wk)						
0	1	<0.001 *	1	0.001 *	1	0.005 *
1–149	0.60 (0.45–0.78)	<0.001	0.63 (0.47–0.83)	0.002	0.62 (0.47–0.81)	0.001
150–299	0.68 (0.45–1.02)	0.060	0.76 (0.50–1.15)	0.194	0.81 (0.54–1.20)	0.294
300-	0.66 (0.54–0.82)	<0.001	0.71 (0.56–0.88)	0.003	0.74 (0.59–0.94)	0.012
Transportation-related physical activity (min/wk)						
0	1	0.060 *	1	0.197 *	1	0.305 *
1–149	0.98 (0.74–1.31)	0.914	1.07 (0.81–1.42)	0.619	1.12 (0.85–1.48)	0.420
150–299	0.51 (0.36–0.74)	<0.001	0.57 (0.38–0.83)	0.004	0.58 (0.40–0.85)	0.006
300-	0.86 (0.59–1.25)	0.429	0.90 (0.61–1.34)	0.611	0.94 (0.63–1.39)	0.740

The multivariable model 1 was adjusted for age, sex, race/ethnicity, education level, marital status, economic status, smoking status, hypertension, diabetes, total cholesterol, total calorie intake, caffeine consumption, and alcohol consumption using appropriate sampling weights. The multivariable model 2 was adjusted for waist circumference in addition to model 1 using appropriate sampling weights. Abbreviations: NAFLD, nonalcoholic fatty liver disease; HR, hazard ratio; CI, confidence interval. * *p*-values were analyzed using the test of the trend of hazards.

Table S10. Multivariable Hazards Ratio for Cardiovascular Mortality based on the Meeting Physical Activity Guideline among Individuals without NAFLD defined by Hepatic Steatosis Index.

	**Age, sex-adjusted model**		**Multivariable model 1**		**Multivariable model 2**	
	**HR (95% CI)**	***p*-value**	**HR (95% CI)**	***p*-value**	**HR (95% CI)**	***p*-value**
Total physical activity						
<150 (min/wk)	1		1		1	
≥150 (min/wk)	0.47 (0.34–0.64)	<0.001	0.53 (0.38–0.73)	<0.001	0.56 (0.40–0.79)	0.001
Leisure-time physical activity						
<150 (min/wk)	1		1		1	
≥150 (min/wk)	0.35 (0.24–0.52)	<0.001	0.38 (0.25–0.57)	<0.001	0.40 (0.27–0.59)	<0.001
Occupation-related physical activity						
<150 (min/wk)	1		1		1	
≥150 (min/wk)	0.72 (0.50–1.03)	0.073	0.86 (0.58–1.26)	0.429	0.91 (0.61–1.35)	0.637
Transportation-related physical activity						
<150 (min/wk)	1		1		1	
≥150 (min/wk)	0.73 (0.43–1.23)	0.230	0.71 (0.40–1.25)	0.233	0.74 (0.41–1.34)	0.310

The multivariable model 1 was adjusted for age, sex, race/ethnicity, education level, marital status, economic status, smoking status, hypertension, diabetes, total cholesterol, total calorie intake, caffeine consumption, and alcohol consumption using appropriate sampling weights. The multivariable model 2 was adjusted for waist circumference in addition to model 1 using appropriate sampling weights. Abbreviations: NAFLD, nonalcoholic fatty liver disease; HR, hazard ratio; CI, confidence interval.

Table S11. Multivariable Hazards Ratio for Cancer-related Mortality based on the Meeting Physical Activity Guideline among Individuals with NAFLD defined by Hepatic Steatosis Index.

	**Age, sex-adjusted model**		**Multivariable model 1**		**Multivariable model 2**	
	**HR (95% CI)**	***p*-value**	**HR (95% CI)**	***p*- value**	**HR (95% CI)**	***p*- value**
Total physical activity						
<150 (min/wk)	1		1		1	
≥150 (min/wk)	0.99 (0.68–1.45)	0.977	1.15 (0.75–1.74)	0.520	1.16 (0.76–1.77)	0.485
Leisure-time physical activity						
<150 (min/wk)	1		1		1	
≥150 (min/wk)	1.02 (0.66–1.57)	0.926	1.13 (0.71–1.78)	0.602	1.16 (0.73–1.82)	0.532
Occupation-related physical activity						
<150 (min/wk)	1		1		1	
≥150 (min/wk)	0.93 (0.61–1.40)	0.708	0.98 (0.63–1.53)	0.938	0.98 (0.63–1.53)	0.943
Transportation-related physical activity						
<150 (min/wk)	1		1		1	
≥150 (min/wk)	0.95 (0.52–1.72)	0.858	0.96 (0.51–1.81)	0.901	0.97 (0.51–1.82)	0.911

The multivariable model 1 was adjusted for age, sex, race/ethnicity, education level, marital status, economic status, smoking status, hypertension, diabetes, total cholesterol, total calorie intake, caffeine consumption, and alcohol consumption using appropriate sampling weights. The multivariable model 2 was adjusted for waist circumference in addition to model 1 using appropriate sampling weights. Abbreviations: NAFLD, nonalcoholic fatty liver disease; HR, hazard ratio; CI, confidence interval.

Table S12. Multivariable Hazards Ratio for Cancer-related Mortality based on the Meeting Physical Activity Guideline among Individuals without NAFLD defined by Hepatic Steatosis Index.

	**Age, sex-adjusted model**		**Multivariable model 1**		**Multivariable model 2**	
	**HR (95% CI)**	***p*-value**	**HR (95% CI)**	***p*- value**	**HR (95% CI)**	***p*- value**
Total physical activity						
<150 (min/wk)	1		1		1	
≥150 (min/wk)	0.50 (0.36–0.69)	<0.001	0.54 (0.38–0.78)	0.001	0.56 (0.39–0.80)	0.002
Leisure-time physical activity						
<150 (min/wk)	1		1		1	
≥150 (min/wk)	0.58 (0.39–0.85)	0.007	0.67 (0.43–1.02)	0.060	0.67 (0.44–1.04)	0.074
Occupation-related physical activity						
<150 (min/wk)	1		1		1	
≥150 (min/wk)	0.80 (0.54–1.16)	0.232	0.79 (0.52–1.21)	0.274	0.81 (0.53–1.25)	0.340
Transportation-related physical activity						
<150 (min/wk)	1		1		1	
≥150 (min/wk)	0.66 (0.38–1.17)	0.154	0.70 (0.39–1.25)	0.220	0.70 (0.39–1.26)	0.233

The multivariable model 1 was adjusted for age, sex, race/ethnicity, education level, marital status, economic status, smoking status, hypertension, diabetes, total cholesterol, total calorie intake, caffeine consumption, and alcohol consumption using appropriate sampling weights. The multivariable model 2 was adjusted for waist circumference in addition to model 1 using appropriate sampling

weights. Abbreviations: NAFLD, nonalcoholic fatty liver disease; HR, hazard ratio; CI, confidence interval.

Table S13. Multivariable Hazards Ratio for All-Cause and Cardiovascular Mortality based on the Amount of Sitting Time among Individuals with NAFLD defined by US Fatty Liver Index.

	Age, sex-adjusted model		Multivariable model 1		Multivariable model 2	
	**OR (95% CI)**	***p*-value**	**OR (95% CI)**	***p*-value**	**OR (95% CI)**	***p*-value**
All-cause mortality						
Sitting time						
Q1 (<4 hr)	1	0.012 *	1	0.056 *	1	0.081 *
Q2 (4-<6 hr)	1.01 (0.67–1.52)	0.967	1.03 (0.70–1.51)	0.872	1.02 (0.69–1.50)	0.915
Q3 (6-<8 hr)	0.92 (0.54–1.58)	0.765	0.92 (0.50–1.68)	0.773	0.90 (0.49–1.67)	0.743
Q4 (≥8 hr)	1.45 (1.05–2.01)	0.024	1.39 (0.98–1.98)	0.065	1.36 (0.95–1.96)	0.095
Total physical activity (min/wk)						
0			1	0.019 *	1	0.025 *
1–149			0.64 (0.46–0.91)	0.014	0.66 (0.47–0.92)	0.016
150–299			0.60 (0.34–1.06)	0.077	0.61 (0.35–1.07)	0.082
300-			0.68 (0.50–0.92)	0.014	0.69 (0.51–0.94)	0.019
Cardiovascular mortality						
Sitting time						
Q1 (<4 hr)	1	0.008 *	1	0.080 *	1	0.119 *
Q2 (4-<6 hr)	0.83 (0.42–1.62)	0.582	0.78 (0.39–1.52)	0.457	0.76 (0.39–1.50)	0.427
Q3 (6-<8 hr)	0.72 (0.26–1.97)	0.514	0.68 (0.24–1.92)	0.460	0.66 (0.24–1.86)	0.428
Q4 (≥8 hr)	1.84 (1.07–3.13)	0.026	1.43 (0.86–2.39)	0.163	1.38 (0.82–2.33)	0.227
Total physical activity (min/wk)						
0			1	0.033 *	1	0.053 *
1–149			0.73 (0.36–1.49)	0.379	0.75 (0.37–1.52)	0.420
150–299			0.52 (0.20–1.34)	0.170	0.53 (0.20–1.43)	0.206
300-			0.54 (0.30–0.97)	0.041	0.56 (0.30–1.03)	0.062

The multivariable model 1 was adjusted for age, sex, race/ethnicity, education level, marital status, economic status, smoking status, hypertension, diabetes, total cholesterol, total physical activity, total calorie intake, caffeine consumption, and alcohol consumption using appropriate sampling weights. The multivariable model 2 was adjusted for waist circumference in addition to model 1 using appropriate sampling weights. Abbreviations: NAFLD, nonalcoholic fatty liver disease; OR, odds ratio; CI, confidence interval. Weighted quartile 1, <4 hours; quartile 2, ≥4 hours to <6 hours; quartile 3, ≥6 hours to <8 hours; quartile 4, ≥8 hours. * *p*-values were analyzed using the test of the trend of hazards.

Table S14. Multivariable Hazards Ratio for All-Cause and Cardiovascular Mortality based on the Amount of Sitting Time among Individuals without NAFLD defined by Hepatic Steatosis Index.

	Age, sex-adjusted model		Multivariable model 1		Multivariable model 2	
	**OR (95% CI)**	***p*-value**	**OR (95% CI)**	***p*-value**	**OR (95% CI)**	***p*-value**
All-cause mortality						
Sitting time						
Q1 (<4 hr)	1	<0.001 *	1	<0.001 *	1	0.001 *
Q2 (4-<6 hr)	1.11 (0.87–1.41)	0.393	1.10 (0.90–1.35)	0.361	1.10 (0.89–1.36)	0.364
Q3 (6-<8 hr)	1.20 (0.94–1.52)	0.137	1.16 (0.92–1.47)	0.216	1.18 (0.93–1.50)	0.160
Q4 (≥8 hr)	1.81 (1.49–2.19)	<0.001	1.51 (1.23–1.86)	<0.001	1.43 (1.16–1.77)	0.001
Total physical activity (min/wk)						
0			1	<0.001 *	1	<0.001 *
1–149			0.76 (0.59–0.97)	0.030	0.78 (0.61–1.01)	0.056
150–299			0.42 (0.33–0.53)	<0.001	0.44 (0.34–0.57)	<0.001
300-			0.47 (0.37–0.59)	<0.001	0.49 (0.39–0.62)	<0.001

Cardiovascular mortality						
Sitting time						
Q1 (<4 hr)	1	<0.001 *	1	0.011 *	1	0.019 *
Q2 (4-<6 hr)	1.30 (0.82–2.05)	0.249	1.34 (0.81–2.21)	0.250	1.30 (0.78–2.16)	0.309
Q3 (6-<8 hr)	1.31 (0.84–2.05)	0.232	1.25 (0.75–2.09)	0.380	1.27 (0.75–2.13)	0.366
Q4 (≥8 hr)	2.47 (1.56–3.90)	<0.001	1.91 (1.15–3.15)	0.013	1.77 (1.08–2.88)	0.023
Total physical activity (min/wk)						
0			1	<0.001 *	1	0.001 *
1–149			0.69 (0.41–1.16)	0.161	0.71 (0.41–1.22)	0.210
150–299			0.56 (0.38–0.82)	0.003	0.59 (0.40–0.87)	0.009
300-			0.49 (0.33–0.72)	<0.001	0.51 (0.34–0.77)	0.002

The multivariable model 1 was adjusted for age, sex, race/ethnicity, education level, marital status, economic status, smoking status, hypertension, diabetes, total cholesterol, total physical activity, total calorie intake, caffeine consumption, and alcohol consumption using appropriate sampling weights. The multivariable model 2 was adjusted for waist circumference in addition to model 1 using appropriate sampling weights. Abbreviations: NAFLD, nonalcoholic fatty liver disease; OR, odds ratio; CI, confidence interval. Weighted quartile 1, <4 hours; quartile 2, ≥4 hours to <6 hours; quartile 3, ≥6 hours to <8 hours; quartile 4, ≥8 hours. * *p*-values were analyzed using the test of the trend of hazards.
